# Influence of Atopic Dermatitis on Cow’s Milk Allergy in Children

**DOI:** 10.3390/medicina55080460

**Published:** 2019-08-10

**Authors:** Arianna Giannetti, Francesca Cipriani, Valentina Indio, Marcella Gallucci, Carlo Caffarelli, Giampaolo Ricci

**Affiliations:** 1 Pediatric Unit, Department of Medical and Surgical Sciences, University of Bologna, 40138 Bologna, Italy; 2 “Giorgio Prodi” Cancer Research Center, University of Bologna, 40138 Bologna, Italy; 3 Clinica Pediatrica, Department of Medicine and Surgery, University of Parma, 43100 Parma, Italy

**Keywords:** atopic dermatitis, children, cow’s milk allergy, food allergy, sensitization, tolerance

## Abstract

*Background and Objectives*: Cow’s milk protein allergy (CMA) is the most common allergy in children. The natural history of CMA is generally favorable and the majority of children reach tolerance during childhood, even if studies show variable results. Atopic dermatitis (AD) is a complex disease from an immunological point of view. It is characterized by an impaired skin barrier function and is often the first clinical manifestation of the so-called “atopic march”. The aim of our study is to evaluate, in a cohort of children with CMA, if the presence of AD in the first months of life can influence the atopic status of patients, the tolerance acquisition to cow’s milk, the level of specific IgE (sIgE), and the sensitization towards food and/or inhalant allergens. *Materials and Methods*: We enrolled 100 children with a diagnosis of CMA referred to our Pediatric Allergology Unit, aged 1–24 months at the time of the first visit. *Results*: 71 children had AD and 29 did not. The mean follow-up was 5.28 years. The CMA manifestations were mainly cutaneous, especially in children with AD (91.6% vs. 51.7%; *P* < 0.001). Patients with AD showed higher rates of polysensitization to foods and higher levels of both total IgE and sIgE for milk, casein, wheat, peanuts, and cat dander at different ages when compared to patients without AD. We analyzed the presence of IgE sensitization for the main foods and inhalants at various ages in the two groups of patients: a statistically significant difference emerged in the two groups of patients for milk, yolk and egg white, hazelnut, peanuts, soybean, grass pollen and cat dander. Meanwhile, we did not find significant differences in terms of tolerance acquisition toward cow’s milk, which was nonetheless reached around 5 years of age in 61% of patients. The level of cow’s milk sIgE at the age of 5 years was significantly higher in the group of patients who did not acquire tolerance (38.38 vs. 5.22 kU/L; *P* < 0.0001). *Conclusions*: An early barrier deficiency appears to promote the development of allergic sensitization, but does not seem to influence the acquisition of tolerance.

## 1. Introduction

Cow’s milk allergy (CMA) may be defined as a reproducible adverse reaction to one or more milk proteins (usually caseins or whey β-lactoglobulin) [1].

CMA is the most common allergy in children with a prevalence between 1.8% and 7.5% during the first year of life [2]. The variability between studies may be attributable to different methods used for diagnosis, the different ages of the populations studied [3] or to geographical factors. This is particularly relevant in CMA as it may appear with a variety of clinical symptoms, many of which can be difficult to attribute to an allergic reaction, particularly in infants [4]. In general, the frequency of self-reported adverse reactions to cow’s milk proteins (CMP) is much higher than the number of medically confirmed diagnoses, not only in children but also in adults [5]. In a Danish cohort of 1749 children [6], followed from birth until the age of 3 years, CMA was suspected in 6.7% of cases and confirmed in 2.2% of cases. In a Norwegian cohort of 3623 children, followed from birth up until the age of 2 years, parents completed questionnaires regarding adverse food reactions at 6 months intervals. In the first phase of the study [7], the cumulative incidence of adverse food reactions was 35% at 2 years of age, and cow milk was the most common food associated with an adverse reaction with an incidence of 11.6%. In the second phase of the study [8], children with persistent CMA or egg allergy at the age of 2 years underwent a more detailed evaluation including skin prick test and open or double-blind oral challenge. At the age of 2.5, the prevalence of allergy and intolerance to milk was 1.1%. Most reactions to milk were not IgE mediated.

Cow’s milk allergy commonly develops early in life, and in almost all cases before 12 months of age [9]. The natural course of cow’s milk allergy is generally favorable, as most children outgrow their allergy during childhood, even if studies show very different ages and rates of resolution [6,10,11,12,13,14,15,16]. Data from literature showed that the presence of immediate symptoms [16,17,18], other food allergies (in particular egg allergy) [16,17,18], concomitant asthma [10,14,16,19] and allergic rhinitis [10,19] can be predictors of tolerance. Food allergy is more common in children with atopic dermatitis, with a proportion of 27.4% for CMA [20]. 

Atopic dermatitis (AD) is the most common chronic skin disease in childhood and it affects 17% to 24% of children and between 4% and 7% of adults [21]. The etiopathogenesis of AD involves a combination of genetic predisposition, impaired skin barrier function, and exposure to environmental triggers. AD is often the first manifestation of the “atopic march”.

The aim of our study is to evaluate in a cohort of children with CMA, if AD in the first months of life can influence the atopic status of patients, the tolerance acquisition to cow’s milk, the level of specific IgE (sIgE) and the sensitization towards food and/or inhalant allergens.

## 2. Materials and Methods

### 2.1. Study Population and Inclusion Criteria

We performed an observational, prospective, real-life study in children diagnosed with CMA consecutively under observation in our Pediatric Allergology Clinic from February 1999 to April 2015. The study was approved by the local Ethics Committee (127/2012/O/Oss, approval date 12/06/2012).

Children were enrolled according to the following criteria:

1. Age included at the time of the first visit between 1 and 24 months;
2. Availability of a detailed personal history and complete clinical evaluation;
3. Performing allergy tests (determination of sIgE for the main food and inhalant allergens and total IgE at 6 months, 7–12 months, 13–24 months, 2–3 years, 3–5 years, and >5 years);and
4. Signed informed consent.

The timing of clinical and allergological evaluations were established according to patients’ clinical needs; therefore, the data available for each age group varied.

### 2.2. Clinical Assessment

Diagnosis of CMA was made in patients with IgE-mediated clinical symptoms after the ingestion of CMP associated with the detection of IgE sensitization to cow’s milk. 

Patients were grouped according to the presence or absence of the AD. Diagnosis of AD was performed by the physician on the basis of the Hanifin and Rajka criteria [22]. First-degree relatives were regarded as atopic if they reported a doctor-diagnosed asthma, rhinoconjunctivitis, AD, and/or oral allergy syndrome/food allergy.

### 2.3. Allergometric Assessment

The determination of sIgE was performed by ImmunoCAP (ThermoFisher Scientific, Uppsala, Sweden) for the main food allergens (cow’s milk, hen’s egg, soybean, wheat, hazelnut, peanuts, and codfish), and the main inhalant allergens (grass pollen, house dust mite *Dermatophagoides pteronyssinus* and *Dermatophagoides* farinae, cat dander, dog dander, wall pellitory, birch, hazel, olive, and *Alternaria*). sIgE levels greater than 0.35 kUa/L were considered as positive. 

Furthermore, the patients were divided into:

- Monosensitized: patients with positive sIgE only to cow’s milk;
- Oligosensitized: patients sensitized to cow’s milk and another allergen; and
- Polysensitized: patients with positive sIgE to cow’s milk and at least two others allergens.

The level of total serum IgE was determined using the ELISA method.

### 2.4. Statistical Methods

Statistical analysis was performed with SPSS Version v23 statistic software package. (IBM Corp. Released 2013. IBM SPSS Statistics for Windows, Version 22.0. Armonk, NY: IBM Corp).

The analysis aimed to study the relation between the presence of AD and several categorical and continuous variables. In particular, a Fisher’s exact test was adopted to test the association between AD status and sensitization to inhalant and food allergens (mono-, oligo-, or polysensitized) at various ages (6 months, 7–12 months, 13–24 months, 2–3 years, 3–5 years, and over 5 years). The same type of statistical test was performed to evaluate the presence/absence of AD depending on the presence of CMA, the first-degree familial atopy, the type of clinical manifestation (cutaneous, gastrointestinal or anaphylaxis) at the diagnosis of CMA, the positivity for food and inhalant allergens, and the presence of asthma and allergy rhinoconjunctivitis. A one-way analysis of variance (ANOVA) was performed to assess the association between sIgE levels (in patients with positive sIgE levels) and the presence of AD at each of the different ages (previously cited) after determining the variances’ homogeneity with the Brown-Forsythe test. In particular, an ANOVA was adopted to study the total IgE level, the sIgE of all the allergens and the milk sIgE at the time patients reached tolerance. For each statistical test, the results were considered significant if the *p*-value was <0.05. 

To evaluate the time courses of milk tolerance acquisition the Kaplan-Maier estimator was adopted followed by a log-rank test to compare the tolerance times in the two group of patients with and without AD.

## 3. Results

One hundred patients diagnosed with CMA were included in the study: 63 males (63%) and 37 females (37%). Seventy-one children (44 males and 27 females) had AD and 29 (19 males and 10 females) did not. The mean follow-up was 5.28 years. The average age of children at first observation was 8.74 months. CMA was diagnosed at an average age of 4.87 months, while AD, in the affected children, was diagnosed at an average age of 4 months.

### 3.1. Familial Atopy

First-degree familial atopy was present in 48 children with AD (in 3 of these there was also a familial history of AD) and in 14 children without AD. No statistically significant differences in familial atopy were found between the two groups. 

### 3.2. Clinical Manifestations

The clinical manifestations of CMA at diagnosis in children with and without AD are shown in Table 1.

In children with AD, CMA occurred more frequently with skin reactions compared to those without this disease (*P* < 0.0001).

### 3.3. IgE Sensitization to Food and Inhalant Allergens

In children with and without AD, the presence of mono-, oligo- and polysensitization to food allergens (Table 2) was evaluated.

The presence of polysensitization to food allergens was significantly higher across all ages in the group of children with AD compared to those who did not have this condition. No statistically significant differences in the IgE sensitization to inhalant allergens were found between the two groups, but patients with AD presented a greater tendency to polysensitization.

The average levels of total IgE and sIgE for the main foods and inhalant allergens at 6 months, 7–12 months, 13–24 months, 2–3 years, 3–5 years and >5 years were compared (Table 3 and Table 4).

The analysis showed that total IgE mean levels were significantly higher in children with AD compared to those without AD at the age of 6 months (129.56 vs. 19.7 UI/mL, *P* < 0.001) and 13–24 months (314.05 vs. 118.39 UI/mL, *P* = 0.017).

Children with AD showed higher levels of sIgE to several foods at different ages: to cow’s milk (sIgE 9.90 vs. 2.34 kU/L; *P* = 0.0034) and casein (sIgE 9.95 vs. 0.59 kU/L; *P* = 0.0014) at the ages of 6 months (Table 3), to wheat at the ages of 7–12 months (sIgE 3.62 vs. 1.43 kU/L; *P* = 0.0011), to peanuts at the age of 7–12 months (sIgE 2.30 vs. 0.43 kU/L, *P* < 0.001), and to cat dander at the ages of 3–5 years (sIgE 2.40 vs. 0.83 kU/L, *P* = 0.00).

In addition, the presence or absence of positivity for the main foods and inhalant allergens at 6 months, 7–12 months, 13–24 months, 2–3 years, 3–5 years and >5 years was evaluated in the two groups of patients. We found a statistically significant difference in the two groups of patients for milk, yolk and egg whites (Table 3), halzenuts, peanuts, soybeans, grass pollen (Table 4), and cat dander.

### 3.4. Atopic Dermatitis (AD) Associated with Asthma and Allergic Rhinoconjunctivitis

In children with AD, asthma occurred in 13 children (18.3%), and in those without AD in 3 children (10.3%). The average age at onset was 7.9 years in the first group and 6 years in the second. Allergic rhinoconjunctivitis occurred in 28 children (39.4%) with AD and in 8 (27.6%) without AD, with no significant difference in the age of onset in the two groups (6.25 years in the group with AD vs. 5.87 in the group without AD).

### 3.5. Tolerance to Cow’s Milk Proteins

**Figure 1:**
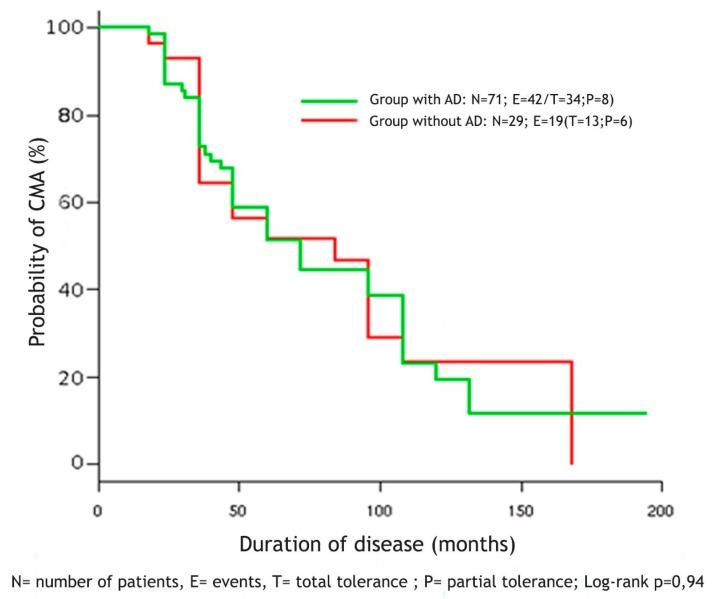
Duration of cow’s milk allergy in the group of patients with and without AD evaluated with Kaplan-Meier analysis.

Among 100 children enrolled in the study, 39 did not achieve tolerance to CMP, 47 achieved complete tolerance and 14 partial tolerance. In Figure 1 we showed the Kaplan-Meier curve on duration of CMA in the two groups of children with and without AD. 

No statistically significant differences emerged in the number of patients nor in the average age of acquisition of tolerance (4.7 years in patients with AD vs. 5 years in those without AD)

**Figure 2:**
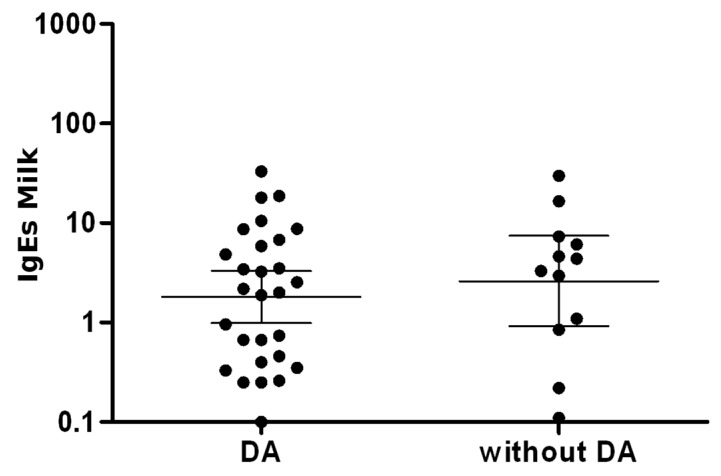
Average level of sIgE to cow’s milk at tolerance acquisition in patients with and without AD.

In 25 patients with AD and in 12 patients without AD, the average level of sIgE to cow’s milk at tolerance acquisition was assessed (4.97 kU/L in patients with AD vs. 6.45 kU/L in patients without AD) (Figure 2). 

**Figure 3:**
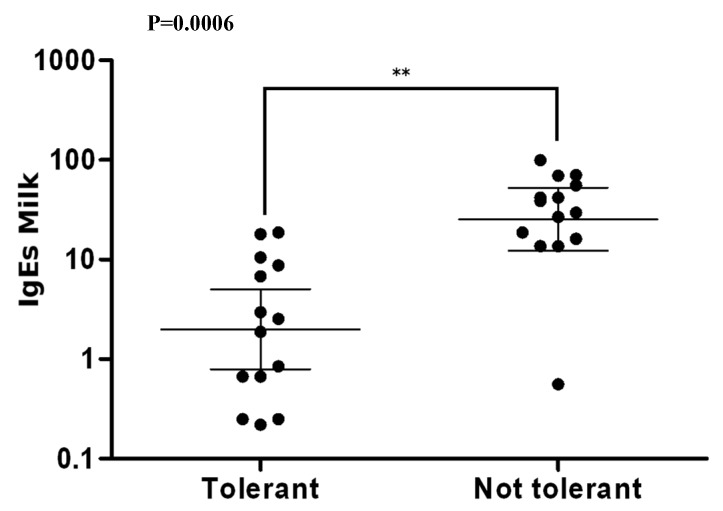
Average level of sIgE to cow’s milk in tolerant and non tolerant patients at 5 years.

The average level of sIgE to cow’s milk at 5 years (average tolerance value in our cohort of children) was then compared. The mean level of sIgE to cow’s milk was significantly higher in the group of non-tolerant children (38.38 vs. 5.22 kU/L; *P* = 0.0006) (Figure 3).

## 4. Discussion

In this study, we evaluated in a prospective, observational, real-life study the influence of comorbid AD in a cohort of children with CMA, consecutively under observation in the Pediatric Allergology Unit.

Seventy-one children out of 100 with CMA were affected by AD, a proportion of children slightly higher than described in the study by Novembre et al. [23], in which almost one-third of children with AD had a diagnosis of CMA based on elimination diet and oral food challenge, and approximatively 40%–50% of children aged <1 year with CMA also had AD.

The manifestations of CMA are mainly cutaneous, especially in children with AD (91.6% vs. 51.7%, *P* < 0.0001). The work of Skripak et al. [10] performed in a cohort of 807 children reported that 85% of children had skin symptoms, 46% had gastrointestinal symptoms and 20% had respiratory symptoms as the first manifestation at CMA onset. In the study by Santos et al. [16], CMA occurred in 91% of cases with skin reactions and in 53% with gastrointestinal symptoms. Similarly, Hill et al. described the following onset symptoms: urticaria in 74% of cases, eczema in 19% of cases, vomiting in 41%, and diarrhea in 33% [1].

### 4.1. Sensitization to Foods

In our study, the presence of polysensitization to food allergens was significantly higher in children with AD. This result is in agreement with data reported by Johansson et al. [24] which showed that children with preschool eczema had an increased risk of polysensitization compared to children without eczema at the age of 4 years (monosensitization: OR, 1.79, 95% CI, 1, 40–2.30, oligosensitization: OR, 2.73; 95% CI, 2.01–3.72; and polysensitization: OR, 7.91; 95% CI, 5.18–12.08); however, in their study, there were no differences regarding sensitization to milk, fish and dust mites. Our data also show that sensitization to egg was significantly higher at 6 months, 7–12 months, 13–24 months, 2–3 years and 3–5 years in children with AD than in those without AD. Hill et al. [25] reported that 64% of children with AD with onset prior to 3 months of age were sensitized to egg and/or milk and/or peanut by considering sIgE values greater than 95% of the positive predictive value. De Benedictis et al. [20] in the EPAAC study performed in 2009 compared the presence of allergic sensitization associated with AD in children aged 12–24 months in 94 cities of 12 countries and detected a greater presence of sensitization in Australia (83%), in the United Kingdom (79%) and in Italy (76%). It also showed high levels of sIgE to egg in each country (53% in the United Kingdom), while milk sensitization was higher in Italy (48%) and peanut sensitization was higher in Australia (45%). Tsakok et al. [26] in a recent review based on sixty-two studies (18 were in the basic population, 8 in high-risk cohorts, and the rest included patients diagnosed with AD or food allergy) shows that, in population-based studies, the probability of food sensitization at 3 months of age was up to 6 times higher in patients with AD compared to healthy controls (OR 6.18, 95% CI, 2.94–12.98, *P* <0.001). Other population studies reported that up to 53% of subjects with AD, were sensitized to foods. In studies including only patients diagnosed with AD the sensitization rate was up to 66%.

### 4.2. Sensitization to Inhalant Allergens

The early onset of AD is often the first clinical manifestation of an allergic disease, which then evolves into the so-called “atopic march” (the subsequent appearance of food allergy, rhinitis and asthma).

In our work, the presence of sensitization to grass pollen was significantly higher in patients with AD, and children with this disease tended to be more polysensitized to inhalants. These data are in agreement with other studies. In a randomized study [27] including 2650 children in Poland, AD was diagnosed in 235 participants (8.9%), 116 children aged 6 to 7 (8.7%) and 119 children aged between 13 and 14 years (9.0%). Skin prick tests for aeroallergens were positive in 1165 children (43.9%): 64.72% of patients with AD compared to 41.9% of patients without AD. The most frequent allergens were: D. pteronyssinus (13.5%), D. farinae (11.7%) and grass pollen (11.8%). In the work of De Benedictis [20] out of 2096 children with AD, the presence of sensitization to at least one inhalant was present in 31.5% of cases, in particular 8.3% were positive for grass pollen and 20.5% for house dust mites.

### 4.3. Specific IgE

In our study we also analyzed the average sIgE level for each allergen in the two groups of patients, showing that the group of patients with AD had higher levels for cow’s milk, casein, wheat, and peanuts with statistically significant differences. The mean total IgE level was also higher in the AD group. High levels of total and/or sIgE undoubtedly represent the most often identifiable biomarkers in subjects with AD, so that their presence or absence makes it possible to distinguish the two main AD phenotypes: the IgE associated form (also called extrinsic) and the non-IgE-associated (or intrinsic) form [28]. In particular, the pattern of sIgE sensitization to different allergens seem to determine the individual profile of each patient [28]. In a recent article [29], Bieber describes the importance of defining biomarkers that allow to distinguish the different phenotypes of AD, also in relation to the underlying genetic bases.

### 4.4. Asthma and Rhinoconjunctivitis

Asthma and rhinoconjunctivitis appeared more frequently in children with AD than in those without AD, even if this difference was not significant (asthma: 18.3% in patients with AD vs. 10.3% in patients without AD; rhinoconjunctivitis: 39.4% in patients with AD vs. 27.6% in patients without AD). This trend confirms that when AD is associated with other allergic conditions, it may represent the first manifestation of the so-called “atopic march”.

Also, in the work of Celakovska et al. [30], AD patients with confirmed food allergy suffered significantly more often from rhinitis and asthma.

Several studies suggest that epicutaneous allergic sensitization could act as a trigger for systemic allergic response involving upper and lower airways. In the work of Lack et al. [31], atopic children exposed to topical emollients containing detectable peanut proteins had an increased risk to develop sensitization to peanuts. Dohi et al. [32] examined the presence of sensitization to dust mite in 8 patients with asthma, but without AD, and 8 patients with AD without asthma. Children with AD had higher levels of sIgE to house dust mites. Both groups underwent provocation tests with acetylcholine, with a non-specific bronchodilator, and with house dust mites. Both groups showed bronchial hypersensitivity to house dust mites, and the response of patients with AD to acetylcholine varied from normal to the asthmatic range. These results indicate that patients with skin sensitization to dust mites may develop symptoms after exposure of the airways to the same allergen.

### 4.5. Natural History of CMA

The natural history of CMA is generally favorable, but studies show different resolution rates. 

Studies performed before 2005 showed a good prognosis for CMA with resolution rates between 70–90% in school age [6,11,12,13,14] while the following studies reported less optimistic data [10,15,16]. This variability can be attributed mainly to methodological differences. In the last three studies, oral food challenge was delayed until the appearance of a reduction of sIgE levels and this may have led to an underestimation of the resolution rate, whereas in previous studies, oral food challenges were performed independent of the sIgE concentration [11,14].

In our study, 61% of children achieved tolerance at an average age of 4.7 years in patients with AD and 5 years in patients without AD.

The AD does not seem to influence the achievement of tolerance, as in our work we did not find a difference between the two groups of patients. The average level of sIgE to cow’s milk at the age of 5 years (mean age at tolerance acquisition in our cohort of children) was then compared between tolerant and non tolerant patients. sIgE was significantly higher in non tolerant patients than in tolerant patients (38.38 vs. 5.22 kU/L).

This result is in agreement with the Fiocchi et al. study [19] in which sIgE to cow’s milk levels were significantly higher in tolerant than in non-tolerant children (20.76 vs. 6.25 kU/L). Petriz et al. [33] in a cohort of 72 children with CMA showed a significant association between the wheal size at skin prick test at the time of diagnosis, casein sensitization and disease persistence. Wood et al. [34] in a cohort of 293 children with CMA identified the main predictors of baseline tolerance in the sIgE level to cow’s milk, wheal size of skin prick test with cow’s milk and severity of AD.

## 5. Conclusions

In conclusion, our study highlights that 71% of children with CMA have a comorbid AD, and CMA in children with AD appears mainly with skin reactions. Children with AD seem to be more polysensitized to food, compared to those without AD, have higher total IgE levels, and have higher levels of sIgE to cat dander and to milk, casein, peanuts and wheat in different age groups. These findings suggest the importance to evaluate, in younger children with CMA and comorbid AD, the presence of IgE sensitization to the most allergenic foods not yet introduced during weaning, in particular to hen’s egg, in order to avoid possible reactions during at-home introduction. On the other hand, no significant differences emerged in the achievement of tolerance to cow’s milk proteins, which was nonetheless, reached in 61% of patients around 5 years of age.

## Figures and Tables

**Table 1:** Clinical manifestations of cow’s milk allergy (CMA) in children with and without atopic dermatitis (AD).

Clinical Manifestations	*n* = 71, AD (+)	*n* = 29, AD (−)	*P*
**Skin**	**65 (91.6%)**	**15 (51.7%)**	**<0.0001**
Urticaria	63	11	
Urticaria and angioedema	2	-	
Urticaria and vomiting	-	4	
**Gastrointestinal**	**3 (4.2%)**	**8 (27.6%)**	
Vomiting and blood streaked stools	3	-	
Vomiting	-	3	
Vomiting and diarrhea	-	1	
Diarrhea and blood streaked stools	-	4	
**Anaphylaxis**	**3 (4.2%)**	**6 (20.7%)**	

**Table 2:** Sensitization to foods in children with and without AD at various ages.

AD (+)			AD (−)			*P*
**6 months**		*n* = 38 (53.5%)	**6 months**		*n* = 7 (24.1%)	
	Mono	6 (15.8%)		Mono	5 (71.4%)	
	Oligo	8 (21.1%)		Oligo	1 (14.3%)	
	Poly	24 (63.2%)		Poly	1 (14.3%)	**0.006**
**7–12 months**		*n* = 57 (80.3%)	**7–12 months**		*n* = 25 (86.2%)	
	Mono	5 (8.8%)		Mono	8 (32%)	
	Oligo	11 (19.3%)		Oligo	11 (44%)	
	Poly	41 (71.9%)		Poly	6 (24%)	**<0.0001**
**13–24 months**		*n* = 40 (56.3%)	**13–24 months**		*n* = 15 (51.7%)	
	Mono	3 (7.5%)		Mono	5 (33.3%)	
	Oligo	3 (7.5%)		Oligo	7 (46.7%)	
	Poly	34 (85%)		Poly	3 (20%)	**<0.0001**
**2–3 years**		*n* = 28 (39.4%)	**2–3 years**		*n* = 13 (44.8%)	
	Mono	2 (7.1%)		Mono	8 (61.5%)	
	Oligo	1 (3.6%)		Oligo	1 (7.7%)	
	Poly	25 (89.3%)		Poly	4 (30.8%)	**<0.0001**
**3–5 years**		*n* = 30 (42.2%)	**3–5 years**		*n* = 12 (41.4%)	
	Mono	5 (16.7%)		Mono	5 (41.7%)	
	Oligo	3 (10%)		Oligo	4 (33.3%)	
	Poly	22 (73.3%)		Poly	3 (25%)	**0.015**
**>5 years**		*n* = 20 (28.2%)	**>5 years**		*n* = 7 (24.1%)	
	Mono	4 (20%)		Mono	4 (57.1%)	
	Oligo	0 (0%)		Oligo	1 (14.3%)	
	Poly	16 (80%)		Poly	2 (28.6%)	**0.026**

**Table 3:** Trend of sIgE to cow’s milk, eggs and presence/absence of positivity for milk and eggs in the groups of children with and without AD at various ages.

Age	AD	Cow’s Milk N/%	Mean sIgE	Egg Yolk N/%	Mean sIgE	Egg White N/%	Mean sIgE
6 months	+	39/39 (100%) *	9.90 **	18/31 (58%) ***	3.33	29/36 (80.5%) °	10.05
	−	6/7 (85.7%)	2.34	0/7 (0%)	<0.35	1/7 (14%)	0.76
7–12 months	+	55/57 (96.5%)	10.89	37/49 (75.5%) ****	2.39	51/57 (89.5%) °°	8.99
	−	24/26 (92.3%)	9.02	9/24 (37.5%)	2.48	13/26 (50%)	4.19
13–24 months	+	40/41 (97.5%)	9.31	27/38 (71%)	3.06	36/40 (90%) °°°	7.11
	−	14/15 (93.3%)	5.74	7/12 (58.3%)	1.78	9/15 (60%)	4.37
2–3 years	+	27/29 (93.1%)	7.81	16/24 (66.6%)	3.08	25/29 (86.2%) °°°°	6.26
	−	12/12 (100%)	6.16	4/11 (36.4%)	5.62	4/12 (33.3%)	5.94
3–5 years	+	26/29 (89.6%)	7.46	15/26 (57.7%)	2.52	24/29 (82.7%) °°°°°	3.92
	−	10/12 (83.3%)	18.10	4/8 (50%)	1.64	6/12 (50%)	4.30
>5 years	+	15/19 (78.9%)	8.73	7/16 (43.7%)	2.38	14/19 (73.7%)	3.10
	−	6/7 (85.7%)	16.44	1/4 (25%)	0.39	3/7 (42.9%)	4.11

* *P* = 0.017; ** *P* = 0.034; *** *P* = 0.005; **** *P* = 0.002; ° *P* = 0.000; °° *P* = 0.000; °°° *P* = 0.010; °°°° *P* = 0.001; °°°°° *P* = 0.031.

**Table 4:** Trend of sIgE to house dust mites, grass pollen, and presence/absence of positivity for house dust mites, and grass pollen in the groups of children with and without AD at various age.

Age	AD	*Dermatophagoides pteronyssinus* N/%	Mean sIgE	*Dermatophagoides farinae* N/%	Mean sIgE	*Phleum pratense* N/%	Mean sIgE	*Cynodon dactylon* N/%	Mean sIgE
6 months	+	3/32 (9.4%)	1.06	3/33 (9.1%)	0.85	3/31 (9.7%)	1.23	2/33 (6.1%)	0.40
	−	0/5 (0%)	<0.35	0/5 (0%)	<0.35	0/5 (0%)	<0.35	0/5 (0%)	<0.35
7–12 months	+	7/48 (14.6%)	1.85	8/51 (15.7%)	1.11	12/50 (24%) *	1.43	8/49 (16.3%) **	1.13
	−	1/21 (4.8%)	9.92	1/21 (4.8%)	3.49	1/22 (4.5%)	0.36	0/21 (0%)	<0.35
13–24 months	+	7/36 (19.4%)	3.02	8/39 (20.5%)	1.53	16/39 (41%)	1.75	10/31 (32.3%) ***	1.78
	−	2/12 (16.7%)	1.35	1/12 (8.3%)	0.95	3/13 (23.1%)	1.30	0/12 (0%)	<0.35
2–3 years	+	9/21 (42.9%)	2.81	7/27 (25.9%)	3.83	15/27 (55.5%) °	4.21	7/17 (41.2%) °°	4.65
	−	2/10 (20%)	3.82	2/11 (18.2%)	4.46	1/12 (8.3%)	0.38	0/9 (0%)	<0.35
3–5 years	+	8/21 (38.1%)	16.54	11/28 (39.3%)	5.83	18/28 (64.3%) °	8.80	12/21 (57.1%) °°°	4.55
	−	4/10 (40%)	1.92	5/12 (41.7%)	1.29	2/12 (16.7%)	2.60	0/9 (0%)	<0.35
>5 years	+	10/12 (83.3%)	16.22	13/19 (68.4%)	13.44	15/19 (78.9%)	24.10	8/13 (61.5%)	22.69
	−	1/3 (66.6%)	42.10	3/7 (42.8%)	7.51	3/6 (50%)	8.12	1/3 (33.3%)	1.78

* *P* = 0.048; ° *P* = 0.006; ** *P* = 0.049*** *P* = 0.025; °° *P* = 0.024; °°° *P* = 0.003.
